# Mechanistic Study of Yibi Decoction in Treating Rheumatoid Arthritis *via* Modulating the JAK2/STAT3 Signaling Pathway

**DOI:** 10.2174/0118715303459997260621164500

**Published:** 2026-07-07

**Authors:** Chuanhong Huang, Shangtong Chen, Yueping Chen, Tian Xia

**Affiliations:** 1 Graduate Department, Guangxi University of Chinese Medicine, Nanning, Guangxi, 530000 China;; 2 Department of Orthopedics and Hand Surgery, Ruikang Hospital affiliated to Guangxi University of Chinese Medicine, Nanning, Guangxi, 530011 China;; 3 College of Orthopedics and Traumatology, Guangxi University of Chinese Medicine, Nanning, Guangxi, 530000 China

**Keywords:** Decoction, tripterygium wilfordii, rheumatoid arthritis, JAK2/STAT3 signaling, IL17/IL23 axis, immune-mediated diseases, network pharmacology

## Abstract

**Introduction/Objective:**

To investigate the therapeutic effects of Yibi Decoction (YBD) in rheumatoid arthritis (RA) and to elucidate the underlying mechanisms, with a particular focus on the JAK/STAT signaling pathway.

**Methods:**

Network pharmacology analysis was employed to predict the bioactive compounds and potential therapeutic targets of Yibi Decoction. A collagen-induced arthritis (CIA) rat model was established to validate the predicted effects *in vivo*. Western blotting, enzyme-linked immunosorbent assay (ELISA), and histopathological analyses were conducted to evaluate alterations in joint tissues and serum inflammatory biomarkers.

**Results:**

Network pharmacology analysis identified 128 bioactive components in YBD, among which quercetin and luteolin were predicted to be key active compounds. In CIA rats, YBD treatment significantly downregulated the expression of IL-6, IL-17, IL-23, JAK2, and STAT3 in ankle joint tissues (*P* < 0.05), and markedly reduced serum levels of anti–cyclic citrullinated peptide (anti-CCP) antibodies and rheumatoid factor (*P* < 0.05). In parallel, the arthritis index was significantly decreased, and histopathological examination revealed substantial attenuation of synovial hyperplasia and cartilage destruction.

**Discussion:**

These findings suggest that YBD exerts anti-rheumatoid arthritis effects through a multi-component, multi-target regulatory mechanism involving inhibition of the JAK/STAT signaling pathway. Quercetin and luteolin may contribute to these effects by suppressing pro-inflammatory cytokine production and ameliorating joint pathological changes.

**Conclusion:**

YBD ameliorates rheumatoid arthritis by suppressing the JAK/STAT signaling pathway and associated inflammatory responses, thereby providing a mechanistic basis for its potential therapeutic application in rheumatoid arthritis.

## 1 INTRODUCTION

Rheumatoid arthritis (RA) is a complex, chronic systemic autoimmune disease characterized by symmetrical polyarticular synovitis, progressive joint and bone destruction, and persistent systemic inflammation [1-4]. The pathogenesis of rheumatoid arthritis is multifaceted, involving dysregulated activation of both innate and adaptive immune responses, amplification of pro-inflammatory cytokine cascades (including IL-6, TNF-α, and IL-17), and hyperproliferation of synovial fibroblasts, which collectively drive tumor-like invasive behavior of the synovium [5, 6]. Currently, the clinical management of rheumatoid arthritis primarily relies on nonsteroidal anti-inflammatory drugs (NSAIDs), glucocorticoids, disease-modifying antirheumatic drugs (DMARDs), and biologic agents, including Janus kinase (JAK) inhibitors such as tofacitinib and TNF-α antagonists [7, 8]. However, long-term use of these medications is associated with significant adverse effects, including gastrointestinal ulceration, osteoporosis, and opportunistic infections, as well as a substantial financial burden. It is estimated that approximately 40% of patients with rheumatoid arthritis discontinue therapy due to adverse drug reactions or insufficient therapeutic efficacy [9]. Therefore, there is an urgent need to develop novel therapeutic strategies that are both highly effective and safe.

Traditional Chinese Medicine (TCM), characterized by principles of holistic regulation and multi-target intervention, offers distinct advantages in the management of rheumatoid arthritis and provides an alternative therapeutic perspective. In recent years, compound Chinese herbal medicines have attracted increasing attention in mechanistic studies of rheumatoid arthritis due to their multi-component and synergistic properties. Previous studies have reported that Guizhi Shaoyao Zhimu Decoction alleviates rheumatoid arthritis by regulating autophagy in pre-osteoclasts *via* the ROS/NF-κB signaling axis [10], while Wutou Decoction ameliorates immune dysregulation in rheumatoid arthritis by modulating the Th17/Treg balance through inhibition of the JAK2/STAT3 signaling pathway [11]. Modern pharmacological studies have identified Tripterygium wilfordii as a commonly used therapeutic agent in Traditional Chinese Medicine for the management of rheumatoid arthritis. Triptolide and glycosides, the principal active constituents of Tripterygium wilfordii, ameliorate rheumatoid arthritis pathology by inhibiting the NF-κB and MAPK signaling pathways, thereby reducing the secretion of pro-inflammatory cytokines, including IL-6 and TNF-α, and inducing apoptosis in synovial cells [12].

The JAK/STAT signaling pathway serves as a pivotal regulatory hub in the pathological progression of rheumatoid arthritis. Upon binding of pro-inflammatory cytokines, such as IL-6 and IL-23, to their cognate membrane receptors, JAK family kinases (including JAK1 and JAK2) undergo phosphorylation and activation. This activation subsequently leads to phosphorylation of STAT3 and STAT5, which then dimerize and translocate into the nucleus [13]. Within the nucleus, activated STAT proteins regulate the transcription of pro-inflammatory genes, such as matrix metalloproteinase-3 (MMP-3) and receptor activator of nuclear factor-κB ligand (RANKL), thereby promoting synovitis, osteoclast activation, and progressive bone erosion. Notably, STAT3 phosphorylation is markedly elevated in the synovial tissues of patients with rheumatoid arthritis and shows a positive correlation with disease activity, as assessed by the Disease Activity Score in 28 joints (DAS28) [14-16]. Currently, although JAK inhibitors such as baricitinib provide rapid symptomatic relief, their clinical utility is limited by an increased risk of infections and a tendency toward disease relapse following treatment discontinuation [17-19].

Yibi Decoction was developed by Professor Li Guiwen, a distinguished senior traditional Chinese medicine physician in China. Over more than 30 years of clinical application, the formula was continuously refined, ultimately resulting in a formulation with *Tripterygium wilfordii* as the main component. The specific composition is as follows: *Tripterygium wilfordii* 30 g, *Astragalus membranaceus *30 g, *Achyranthes bidentata* 20 g, *Glycyrrhiza uralensis* 20 g. This decoction has achieved good clinical efficacy and can effectively control the progression of rheumatoid arthritis. At present, the synergistic regulatory mechanisms by which compound Chinese herbal medicines modulate the JAK/STAT signaling pathway remain incompletely understood. In particular, the interactions among multiple bioactive constituents and their network-based modes of regulation warrant further in-depth investigation. Accordingly, the present study employed a network pharmacology approach in combination with a collagen-induced arthritis (CIA) rat model, together with Western blotting and enzyme-linked immunosorbent assay (ELISA) analyses, to elucidate the regulatory effects of Yibi Decoction (YBD) on the JAK/STAT signaling pathway and its therapeutic mechanisms in rheumatoid arthritis.

## 2 MATERIALS AND METHODS

### 2.1 Research Design

This study was designed as an integrated experimental investigation combining network pharmacology–based prediction with *in vivo* validation in an animal model to systematically elucidate the therapeutic mechanisms of Yibi Decoction (YBD) in rheumatoid arthritis.

### 2.2 Network Pharmacology

#### 2.2.1 Data Collection and Preparation

Rheumatoid arthritis–associated datasets were retrieved from the Gene Expression Omnibus (GEO) database (https://www.ncbi.nlm.nih.gov/geo/, accessed on 8 March, 2025). Eligible disease-related gene expression datasets were selected according to the following criteria: (1) inclusion of both rheumatoid arthritis patients and normal control samples; (2) a minimum sample size of eight to ensure analytical reliability; and (3) generation based on high-throughput sequencing platforms. A search of the GEO database using the keyword “rheumatoid arthritis” identified three gene expression datasets that met the inclusion criteria for this study: GSE55235, GSE55457, and GSE77298. These datasets were subsequently merged using R software, and batch effects inherent to the high-dimensional data were addressed by applying the “sva” package to estimate and adjust for surrogate variables.

#### 2.2.2 RA Difference Analysis

Following data preprocessing, differential expression analysis was conducted using the Linear Models for Microarray Data (LIMMA) package (version 4.2.2) in R software. The LIMMA package was applied to identify differentially expressed genes (DEGs) between the rheumatoid arthritis (RA) samples and normal control samples. Genes meeting the criteria of an adjusted *P* value < 0.05 and an absolute log_2_ fold change (|log_2_FC|) > 0.4 were considered statistically significant DEGs. Subsequently, heatmaps and volcano plots were generated to visualize the differential expression patterns, with blue and red indicating downregulated and upregulated genes, respectively.

#### 2.2.3 Screen for the Active Ingredients and Pharmacodynamic Targets of the Drug

Compound profiles of *Glycyrrhiza uralensis, Achyranthes bidentata, Astragalus membranaceus*, and *Tripterygium wilfordii* were retrieved from the Traditional Chinese Medicine Systems Pharmacology Database (TCMSP; https://tcmsp-e.com/index.php/, accessed on 9 March, 2025) and PubMed to identify bioactive constituents relevant to the pathogenesis and treatment of rheumatoid arthritis. Potential active compounds were screened based on oral bioavailability (OB) ≥ 30% and drug-likeness (DL) ≥ 0.18. The corresponding target proteins of the identified compounds were subsequently collected, and target gene names were standardized using the UniProt database.

#### 2.2.4 Venn Diagram Screening for Targets of RA Treatment

Intersecting genes between the differentially expressed targets and the herb-derived compound targets were identified using the Jvenn platform (https://jvenn.toulouse.inrae.fr/app/example.html/, accessed on 10 March, 2025), representing putative therapeutic targets for rheumatoid arthritis. Subsequently, a herb–compound–target interaction network was constructed and visualized using Cytoscape software (version 3.9.1).

#### 2.2.5 Core Gene Preselection and Functional Enrichment Analysis

The overlap between predicted drug targets and differentially expressed genes (DEGs) was analyzed to identify key therapeutic targets. To further elucidate the biological functions and associated signaling pathways of these targets, Gene Ontology (GO) functional annotation and Kyoto Encyclopedia of Genes and Genomes (KEGG) pathway enrichment analyses were conducted using the clusterProfiler package in R. The bar chart illustrates that the functional enrichment results were statistically significant at *P* < 0.05. Furthermore, the top 10 significantly enriched Gene Ontology (GO) terms—including those from the Biological Process (BP), Molecular Function (MF), and Cellular Component (CC) categories—as well as the top 30 significantly enriched Kyoto Encyclopedia of Genes and Genomes (KEGG) pathways, were visualized to highlight the most relevant biological features.

### 2.3 Animal Experiment

#### 2.3.1 Experimental Animal

Forty 8-week-old specific pathogen-free female Sprague–Dawley rats (180–210 g) were obtained from Beijing Sipeifu Biotechnology Co., Ltd. (SCXK (Jing) 2019-0010). Animals were housed under controlled conditions (20–26°C, 30–70% humidity, 14 h/12 h light–dark cycle) with ad libitum access to food and water in the SPF facility at Guangxi University of Chinese Medicine. All procedures were approved by the Institutional Animal Welfare and Ethics Committee (Approval No. DW20240919-241) and conducted in accordance with NIH guidelines.

#### 2.3.2 Experimental Agents

The Chinese herbal materials were obtained from the pharmacy of Ruikang Hospital, affiliated with Guangxi University of Chinese Medicine. Baricitinib tablets (HJ20215001; Lilly del Caribe, Inc.) and methotrexate tablets (H31020644; Shanghai Shangyao Xinyi Pharmaceutical Co., Ltd.) were purchased from Sinopharm Group Guangxi Co., Ltd.

#### 2.3.3 Reagents and Instruments

Major reagents included bovine type II collagen (Shanghai Yuanye), Complete and Incomplete Freund’s Adjuvant (Servicebio), RIPA lysis buffer, 5× SDS–PAGE loading buffer, 10% Tris-SwePAGE precast gels, PMSF, 10× TBST (Servicebio), 0.45-µm PVDF membranes (Biosharp), BCA protein quantification kit (Biosharp), and ECL substrate (Biosharp). Primary antibodies against IL-6, IL-17A, IL-23A, JAK2, and STAT3 (all from Solarbio) were used, with HRP-conjugated goat anti-rat IgG (Solarbio) as secondary antibody. Serum levels of cyclic citrullinated peptide (CCP) and rheumatoid factor (RF) were measured using commercial ELISA kits (Jingmei Biotechnology). Key instruments included a refrigerated centrifuge (Eppendorf), a multimode microplate reader (SpectraMax), an electrophoresis system (Liuyi), a gel imaging system (Tanon), a high- throughput cryogenic tissue grinder (Servicebio), and a water purification system (Fulham).

#### 2.3.4 Experimental Animal Grouping and CIA Model Preparation

Rats were randomly assigned to five groups (n = 8 per group) using a random number table: Blank, Model, Methotrexate, Baricitinib, and Yibi Decoction. Sample size was determined by a priori power analysis (G*Power 3.1, one-way ANOVA, α = 0.05, power = 0.80), which indicated a minimum of seven rats per group; eight were used to account for an anticipated 15% attrition rate. The study protocol was approved by the Animal Welfare Ethics Committee of Guangxi University of Chinese Medicine (No. DW20240919-241). After a 1-week acclimatization, CIA was induced in all groups except the Blank group by immunization with bovine type II collagen emulsified in Complete Freund’s Adjuvant (primary) and Incomplete Freund’s Adjuvant (booster).

#### 2.3.5 Preparation of Reagents

Emulsions were prepared on ice. Bovine type II collagen (40 mg) was dissolved in 3.6% (v/v) dilute acetic acid to obtain a uniform suspension, then mixed with an equal volume of Complete Freund’s Adjuvant and emulsified to form a stable water-in-oil emulsion. Emulsification was confirmed by a water-droplet test. The final collagen concentration was 1 mg/mL. Incomplete Freund’s Adjuvant emulsions were prepared using the same procedure.

#### 2.3.6 Injection of Immunological Adjuvant

On day 1, the CFA emulsion was administered *via* subcutaneous injections (0.05 mL/site) at the bilateral hind paw pads and dorsal sites. Booster immunizations with the IFA emulsion (same dosage) were given subcutaneously along the dorsal region on days 7, 14, and 21, with injection sites rotated to mimic the chronic progressive nature of rheumatoid arthritis.

#### 2.3.7 Evaluation of Model Construction Effect

On day 28, arthritis severity in CIA rats was assessed using an Arthritis Index (AI) scoring system (0–4 per paw; total score 0–16). A total score ≥ 8 was considered indicative of successful model establishment. To validate the model, one rat per group was randomly selected for further evaluation: bilateral ankle joints were collected for HE stain, and serum levels of CCP antibodies and RF were measured by ELISA.

#### 2.3.8 Pharmacological Gavage Protocol

Methotrexate (0.8 mg/kg) was administered by oral gavage once weekly. Baricitinib (0.32 mg/kg) was given once daily by oral gavage. Yibi Decoction (YBD) was prepared as a concentrated decoction (5.0 g crude herb/mL) and administered once daily at 3 mL per rat (15 g crude herb/kg). All treatments lasted 4 weeks. The blank control group received an equal volume (3 mL) of purified water daily.

#### 2.3.9 Sample Collection

Sample collection was performed on days 28 and 56. On D28, one rat per group was randomly selected for interim sampling; the remaining rats were sampled on D56. Body weight was recorded before collection. Approximately 4 mL of blood was collected from the abdominal aorta and centrifuged at 3,000 rpm for 15 min. The serum was then stored at −80°C. After blood collection, rats were euthanized by cervical dislocation. One ankle joint was fixed in 4% paraformaldehyde for histopathology, and the contralateral joint was snap-frozen in liquid nitrogen and stored at −80°C for molecular analysis.

#### 2.3.10 Western Blot

Western blotting was used to detect protein expression of JAK2, STAT3, IL-6, IL-17, and IL-23 in rat ankle joint tissues. Briefly, tissue samples were homogenized in lysis buffer (1:10, w/v) using a pre-chilled high-throughput tissue grinder. Homogenates were centrifuged at 12,000 rpm for 7 min at 4°C, and the supernatants were collected. Protein concentrations were determined using a BCA assay. Equal amounts of protein were separated on 10% SDS–PAGE precast gels and transferred onto PVDF membranes. Membranes were blocked with 5% skim milk, incubated with primary antibodies overnight at 4°C, and then with HRP-conjugated secondary antibodies for 2 h at room temperature. Protein bands were visualized using an ECL detection system and captured with a gel imaging system. Band intensities were quantified using ImageJ software.

#### 2.3.11 ELISA

ELISA was performed to measure serum levels of CCP antibodies and RF using commercial kits (Jingmei Biotechnology). Briefly, serum samples and standards were added to designated wells and incubated at 37°C for 30 min. After washing, the enzyme-conjugated reagent was added and incubated for an additional 30 min at 37°C. Chromogenic substrate was then added and incubated for 10 min in the dark. The reaction was terminated, and the optical density was measured immediately using a microplate reader.

#### 2.3.12 HE Stain

Ankle joints were fixed in 4% paraformaldehyde for 48 h, decalcified with 10% EDTA, and then sent to Wuhan Servicebio Technology for paraffin embedding, HE stain, and histopathological evaluation.

## 3 STATISTICAL METHODS

Statistical analyses were performed using SPSS version 27.0 (IBM, USA), and graphical representations were generated using GraphPad Prism version 10.1 (GraphPad Software, USA). Quantitative data are presented as the mean ± standard deviation (SD). Comparisons among multiple groups were conducted using one-way analysis of variance (ANOVA), followed by Bonferroni post hoc tests for pairwise comparisons. A two-tailed *P* value < 0.05 was considered statistically significant.

## 4 RESULT

### 4.1 Network Pharmacology

#### 4.1.1 Batch Effect Elimination in GEO

Preliminary data preprocessing was performed on the three datasets, including quality control (QC) assessments to identify and remove aberrant samples or probes, thereby ensuring data reliability. Background correction was subsequently applied to each dataset to normalize signal intensities and minimize technical variation. Subsequently, appropriate data transformation methods were applied based on dataset characteristics to satisfy the statistical assumptions required for downstream analyses. Consistent probe or gene nomenclature was ensured across all datasets to facilitate accurate matching and integration. Each sample was annotated with a batch label corresponding to its source dataset to facilitate batch effect correction [20]. The ComBat algorithm from the sva package, which is based on an empirical Bayes framework, was applied to adjust for systematic inter-batch variability. The integrated dataset was subsequently corrected using ComBat to minimize non-biological variation attributable to batch effects. Comparisons before and after batch correction were performed to visualize and evaluate the effectiveness of batch effect removal (Figs. **1** and **2**).

#### 4.1.2 DEGs and targets of RA

Using the limma package, a total of 2,495 differentially expressed genes (DEGs) associated with rheumatoid arthritis were identified, including 1,195 downregulated and 1,300 upregulated genes. These DEGs were visualized using a volcano plot (Fig. **3**) and a heatmap displaying the top 40 most statistically significant DEGs (Fig. **4**), both generated with R software (version 4.2.2). Subsequently, all identified DEGs were annotated and standardized against the UniProt database using Perl scripts, yielding 2,495 refined rheumatoid arthritis–related disease targets.

Blue and red indicate downregulation and upregulation, respectively, in both graphical representations.

#### 4.1.3 Key Targets and Regulatory Networks for RA Treatment

Based on the predefined screening criteria, a total of 128 bioactive compounds were identified from the prescribed herbs, including 12 from *Achyranthes bidentata*, 30 from *Tripterygium wilfordii*, 1 from *Astragalus membranaceus*, and 85 from *Glycyrrhiza uralensis*. In addition, 251 putative therapeutic targets associated with these compounds were collected. Intersection analysis between these compound-related targets and the 2,495 rheumatoid arthritis–related disease targets, performed using a Venn diagram, identified 76 overlapping targets (Fig. **5**). Subsequently, a herb–compound–target regulatory network was constructed and visualized, incorporating the four herbs, 128 bioactive compounds, and 76 overlapping targets (Fig. **6**). The resulting network comprised a total of 383 nodes. Network topology analysis revealed that PPARG, CCNA2, CHEK1, CA2, and IGHG1 exhibited the highest degree values among target nodes, while quercetin, luteolin, and β-sitosterol were identified as the most highly connected bioactive compounds.

#### 4.1.4 Functions and Pathways Involved in the Drug Therapy of RA Targets

GO functional enrichment analysis identified 1,621 significantly enriched terms, of which 1,454 were classified under the Biological Process (BP) category. These BP terms were predominantly associated with responses to reactive oxygen species, lipopolysaccharide, ultraviolet A (UV-A) radiation, oxidative stress, abiotic stimuli, and chemical stress, as well as regulation of neuroinflammatory responses. These enriched functions were closely associated with key pathological processes involved in rheumatoid arthritis, including inflammation, oxidative stress, tissue damage and repair, and immune regulation. In addition, 27 significantly enriched (CC) terms were identified, primarily involving membrane rafts, the external side of the plasma membrane, membrane microdomains, low-density lipoprotein particles, neuronal cell bodies, and banded collagen fibers. MF enrichment analysis identified 140 significantly enriched terms, including histone kinase activity, nuclear receptor activity, ligand-activated transcription factor activity, viral receptor activity, cytokine receptor binding, exogenous protein binding, insulin receptor substrate binding, and protein kinase regulatory activity, among others (Fig. **7**). KEGG pathway enrichment analysis identified 113 significantly enriched pathways, primarily including type II diabetes mellitus, the p53 signaling pathway, lipid metabolism and atherosclerosis, rheumatoid arthritis, the IL-17 signaling pathway, Th17 cell differentiation, the chemokine signaling pathway, the Toll- like receptor signaling pathway, and fluid shear stress and atherosclerosis (Fig. **8**).

### 4.2 Animal Experiment

#### 4.2.1 The ELISA Method was used to Evaluate the Modeling Effect

Serum levels of CCP antibodies and RF on D28 were measured using ELISA. Compared with the blank group, the model, methotrexate, baricitinib, and Yibi Decoction groups exhibited significantly elevated serum levels of CCP and RF (*P* < 0.001; Fig. **9**).

#### 4.2.2 Evaluation of the Modeling Effect by HE Staining

One ankle joint tissue sample collected on D28 was paraffin-embedded and subjected to HE staining for histomorphological evaluation. Histopathological examination revealed that rats in the model, methotrexate, baricitinib, and Yibi Decoction groups exhibited characteristic rheumatoid arthritis–associated pathological changes, including cartilage surface destruction, pannus formation, synovial hyperplasia, pronounced neovascularization, and inflammatory cell infiltration (Fig. **10**).

#### 4.2.3 Evaluation of the Modeling Effect by the AI Index

On day 28, AI scores were evaluated for both the fore and hind paws of all rats. The model, methotrexate, baricitinib, and Yibi Decoction groups all exhibited AI scores ≥ 8, with no statistically significant differences observed among these groups (*P* > 0.05). In contrast, AI scores in each of these groups were significantly higher than those in the blank control group (*P* < 0.05) (Fig. **11**; Table **1**).

#### 4.2.4 Western Blot

Following pharmacological intervention, the methotrexate, baricitinib, and Yibi Decoction groups exhibited statistically significant reductions in the expression levels of IL-6, IL-17, IL-23, JAK2, and STAT3 to varying extents compared with the model group (*P* < 0.05) (Fig. **12**).

#### 4.2.5 ELISA

Following pharmacological intervention, serum levels of cyclic citrullinated peptide (CCP) antibodies and rheumatoid factor (RF) were significantly reduced to varying extents in the methotrexate, baricitinib, and Yibi Decoction groups compared with the model group (*P* < 0.05) (Fig. **13**).

#### 4.2.6 Morphological Presentation of Rat Paw After Yibi Decoction Intervention

Following intervention, rats in the Yibi Decoction group exhibited a marked reduction in paw swelling, with skin coloration approaching normal; however, residual deformities of the interphalangeal joints remained evident (Fig. **14**).

HE staining revealed that, before treatment, ankle joint tissues displayed extensive cartilage destruction, pannus formation, pronounced synovial connective tissue hyperplasia, and dense infiltration of inflammatory cells, including lymphocytes and neutrophils. After treatment with Yibi Decoction, the cartilage surface was largely preserved, although disrupted tidemark integrity and focal nuclear dissolution were observed. In addition, only mild hyperplasia of the synovial lining layer and limited focal lymphocytic infiltration were present (Fig. **15**).

## 5 DISCUSSION

The primary pathological features of rheumatoid arthritis include synovial hyperplasia, infiltration of inflammatory cells, and pannus formation—all of which collectively lead to progressive erosion of cartilage and bone [21-23]. A hallmark pathological feature of rheumatoid arthritis is the aberrant activation of fibroblast-like synoviocytes (FLS), which acquire aggressive, tumor-like proliferative and invasive properties [23]. Persistent synovitis leads to progressive destruction of articular cartilage and loss of joint structural integrity, ultimately resulting in clinical manifestations such as joint swelling, pain, and deformity [24]. Rheumatoid arthritis is primarily driven by CD4^+^ T lymphocytes, while B lymphocytes play a crucial role in mediating and sustaining the pathological process [25-27]. In addition, multiple immune and stromal cell types collectively contribute to synovial inflammation and progressive cartilage and bone erosion [28-31]. Notably, IL-6 serves as a critical mediator of bone destruction in rheumatoid arthritis by promoting T-lymphocyte differentiation and activation and amplifying downstream inflammatory responses [32-34]. Rheumatoid arthritis primarily manifests through two major pathological mechanisms. First, aberrant proliferation of synovial tissue is driven by heightened activation of macrophage-like synoviocytes (MLS) and FLS, which secrete large amounts of pro-inflammatory cytokines and matrix-degrading proteases, including IL-1, IL-6, and TNF-α [35]. In addition to expressing IL-6, FLS produce substantial levels of matrix metalloproteinases (MMPs) and lipid-derived inflammatory mediators, such as prostaglandins and leukotrienes. Moreover, FLS actively participate in immune activation by interacting with infiltrating immune cells and promoting the formation of ectopic lymphoid-like structures (ELS) within synovial tissue. The second pathological mechanism involves infiltration of adaptive immune cells into the synovial sublining, leading to the formation of characteristic pannus tissue at the cartilage–bone interface [36-38]. This pannus is composed primarily of macrophages, FLS, dendritic cells, plasma cells, and mast cells, which collectively mediate progressive tissue damage and bone erosion during the later stages of disease [39]. During the early phase of RA, IL-23 promotes the differentiation and stabilization of Th17 cells, which subsequently produce IL-17 and IL-6. IL-17 further stimulates macrophages to generate pro-inflammatory mediators, including TNF-α. In fibroblast-like synoviocytes derived from RA patients, IL-17 upregulates the expression of IL-23p19 at both the mRNA and protein levels, thereby reinforcing the IL-23/Th17 inflammatory axis. This sustained inflammatory loop contributes to B-cell activation and autoantibody production, including RF and anti-citrullinated protein antibodies (ACPAs) [40, 41]. The JAK–STAT signaling pathway is a central mechanism of intracellular signal transduction that conveys extracellular cues from cell-surface receptors to the nucleus, thereby regulating key physiological processes such as cell proliferation, differentiation, and apoptosis. A distinctive feature of the JAK–STAT signaling pathway is its reliance on two core molecular components- Janus kinases (JAKs) and signal transducers and activators of transcription (STATs)—to mediate signal transmission, in contrast to many immune signaling pathways that involve more complex, multi-step cascades [14]. This pathway plays a critical role in a wide range of physiological and pathological processes, including immune regulation, inflammation, neurodegenerative disorders, and oncogenesis. Dysregulation of the JAK–STAT signaling pathway can result in severe immunodeficiency as well as uncontrolled cell proliferation and malignant transformation [42]. During the pathogenesis of rheumatoid arthritis, the JAK/STAT signaling pathway remains chronically activated as a result of impaired negative regulatory mechanisms. This persistent activation leads to markedly elevated production of multiple pro-inflammatory cytokines, including TNF-α, IL-1, IL-6, IL-15, IL-17, IL-18, IL-22, IL-23, IL-32, and granulocyte–macrophage colony-stimulating factor (GM-CSF) [43]. These cytokines, in turn, further enhance JAK/STAT signaling, thereby establishing a self-amplifying positive feedback loop that sustains chronic inflammation. Consequently, this synergistic inflammatory interplay drives the progression of synovitis, wherein hyperplastic and hyperactivated synovial tissue not only facilitates extensive inflammatory cell infiltration but also promotes excessive secretion of matrix-degrading enzymes, increased production of vascular endothelial growth factor (VEGF), and pronounced pannus formation. Together, these pathological alterations establish a self-perpetuating vicious cycle that sustains and exacerbates the progression of rheumatoid arthritis [42]. Sustained activation of the JAK–STAT signaling pathway within the synovium promotes the release of multiple pro-inflammatory cytokines, which in turn stimulate the production of MMPs [44]. These enzymes facilitate osteoclast differentiation and activation, ultimately leading to progressive destruction of cartilage and bone tissues and thereby exacerbating joint pain and swelling [45]. Furthermore, autophagy in fibroblast-like synoviocytes (FLS) from patients with rheumatoid arthritis is constitutively activated, resulting in increased production of receptor activator of nuclear factor kappa-B ligand (RANKL). RANKL binds to its cognate receptor, receptor activator of nuclear factor kappa-B (RANK), on the surface of osteoclast precursors, thereby promoting osteoclast differentiation and bone resorption. Collectively, these pathological processes drive progressive joint bone erosion and destruction and represent a critical mechanism underlying the exacerbation of chronic inflammation and joint damage in rheumatoid arthritis [46-48].

Using the limma package, we identified a total of 2,495 DEGs associated with rheumatoid arthritis, including 1,195 downregulated and 1,300 upregulated genes. These DEGs reflect substantial transcriptomic alterations during RA pathogenesis and provide valuable insights into the underlying molecular mechanisms. Functional characterization indicated that upregulated genes were predominantly associated with inflammatory responses, immune activation, and cell proliferation, whereas downregulated genes were mainly involved in apoptosis, tissue repair, and immune regulatory processes. Among these DEGs, several genes were closely linked to key pathological features of RA. Notably, PPARG was identified as a high-degree target in the network analysis. PPARG is a nuclear receptor that plays an important role in regulating adipocyte differentiation, glucose homeostasis, and inflammatory responses, and has been increasingly recognized as a critical modulator of immune-mediated inflammation in rheumatoid arthritis [49]. In patients with rheumatoid arthritis, activation of PPARG may attenuate joint inflammation by suppressing the production of pro-inflammatory cytokines [50]. Checkpoint kinase 1 (CHEK1) was also identified as a key target in this study. CHEK1 plays a critical role in cell-cycle regulation and DNA damage response, and its aberrant upregulation in rheumatoid arthritis may be associated with sustained cellular proliferation and enhanced inflammatory activity within synovial tissue [51]. A total of 128 bioactive compounds were identified from the selected Chinese medicinal herbs, including *Glycyrrhiza uralensis, Achyranthes bidentata, Astragalus membranaceus*, and *Tripterygium wilfordii*. These herbs have been widely used in traditional medicine for the treatment of rheumatoid arthritis, and their bioactive constituents are thought to exert therapeutic effects through anti-inflammatory, immunomodulatory, and tissue-protective mechanisms. Among the identified compounds, quercetin and luteolin were highlighted as key flavonoids with high abundance, particularly in *Glycyrrhiza uralensis* and *Achyranthes bidentata*. Quercetin has been extensively reported to possess potent anti-inflammatory and antioxidant properties, including the inhibition of pro-inflammatory cytokines such as TNF-α and IL-6 [52-56].

Consistent with the reported pharmacological activities of the identified compounds, Gene Ontology (GO) functional enrichment analysis demonstrated that rheumatoid arthritis–related differentially expressed genes were predominantly involved in biological processes associated with inflammatory responses, oxidative stress, immune regulation, and tissue repair. These enriched functions closely align with the core pathological features of rheumatoid arthritis, supporting the potential relevance of the identified bioactive compounds in modulating disease-associated molecular pathways.

Inflammation represents a central pathological process in RA and is characterized by sustained activation of multiple cytokines and chemokines. Notably, the enrichment of inflammation-related processes, such as the response to lipopolysaccharide, indicates persistent inflammatory stimulation in RA patients. In addition, oxidative stress plays a critical role in RA pathogenesis by exacerbating synovial inflammation and contributing to progressive joint damage [57]. The enrichment of oxidative stress–related biological processes identified in this study, such as the response to ultraviolet A (UV-A) radiation, further supports the critical involvement of oxidative stress in rheumatoid arthritis pathogenesis. In addition, KEGG pathway enrichment analysis demonstrated that RA-related differentially expressed genes were significantly associated with several key signaling pathways, including the p53 signaling pathway, IL-17 signaling pathway, and Toll-like receptor signaling pathway. These pathways are known to play essential roles in regulating inflammatory responses and immune homeostasis in RA. Notably, the p53 signaling pathway, which is central to cell- cycle control and apoptosis, may contribute to pathological features of RA when dysregulated, such as sustained proliferation of inflammatory cells and resistance to apoptosis within synovial tissue [58-60]. The IL-17 signaling pathway plays a central role in the inflammatory pathology of rheumatoid arthritis by inducing the production of multiple pro-inflammatory cytokines that drive joint inflammation and tissue destruction [61-63]. In addition, the Toll-like receptor signaling pathway contributes substantially to immune dysregulation in RA, as its activation promotes inflammatory cell activation and robust cytokine release, thereby amplifying inflammatory responses [64-66]. These bioinformatic findings provide insight into the potential molecular mechanisms by which Chinese herbal medicine may exert therapeutic effects in rheumatoid arthritis [67]. Specifically, the bioactive components of Chinese herbs may confer anti-inflammatory, immunomodulatory, and tissue-repair effects through interactions with RA-related targets, thereby modulating multiple disease-relevant biological processes and signaling pathways. Although the four herbs examined in this study were not directly associated with the JAK/STAT signaling pathway in the prediction analysis, key bioactive compounds such as quercetin, luteolin, and β-sitosterol may modulate this pathway indirectly by interacting with upstream or downstream regulatory targets.

Specifically, quercetin exhibits significant anti-inflammatory and antioxidant activities, capable of inhibiting the production of inflammatory cytokines such as TNF-α and IL-6. In addition, quercetin can alleviate oxidative stress damage by modulating the Nrf2 signaling pathway. Luteolin, on the other hand, inhibits NF-κB activation by preventing IκBα degradation induced by LPS stimulation and blocking the nuclear translocation of the NF-κB p65 subunit, thereby suppressing the NF-κB signaling pathway and inhibiting inflammatory responses [68-70]. Through these interactions, these compounds may inhibit inflammatory cytokine production or attenuate signal transduction, thereby contributing to the alleviation of rheumatoid arthritis symptoms. These findings suggest that the herbal composition of Yibi Decoction may indirectly modulate the JAK/STAT signaling pathway, thereby contributing to its therapeutic effects against rheumatoid arthritis. Beyond indirect regulation of the JAK/STAT axis, Yibi Decoction may also exert its anti-RA effects through additional molecular targets and signaling pathways. For example, bioactive compounds derived from *Glycyrrhiza uralensis* may activate the PPARG signaling pathway, leading to suppression of inflammatory responses and immune activation [39]. Furthermore, herbal constituents may attenuate oxidative damage in patients with rheumatoid arthritis by modulating oxidative stress–related pathways. The multi-target mechanisms of these botanical compounds may confer unique therapeutic advantages in RA management. Compared with conventional single-target drugs, Chinese herbal ingredients can regulate RA-associated pathological processes through multiple signaling pathways, potentially reducing the risks of drug resistance and adverse effects. Notably, multiple compounds within herbal formulations may act synergistically to enhance anti-inflammatory efficacy. This multi-target, multi-pathway therapeutic strategy offers a promising and complementary approach for the treatment of rheumatoid arthritis. Although network pharmacology predicted multiple potential targets and pathways, the present *in vivo* study primarily focused on validating the JAK2/STAT3 axis, and additional targets remain to be experimentally verified.

ELISA results (Fig. **13**) demonstrated that serum levels of CCP antibodies and RF were significantly reduced in the methotrexate, baricitinib, and Yibi Decoction groups following intervention compared with the model group (*P* < 0.05). Notably, the magnitude of reduction observed in the Yibi Decoction group was comparable to that achieved with methotrexate, suggesting that Yibi Decoction exerts a therapeutic effect of similar efficacy in attenuating immune activation associated with rheumatoid arthritis. Furthermore, Western blot analysis demonstrated that the expression levels of IL-6, IL-17, IL-23, JAK2, and STAT3 were significantly reduced in the methotrexate, baricitinib, and Yibi Decoction groups compared with the model group (*P* < 0.05).

In this study, Western blot analysis revealed that YBD intervention significantly reduced the protein expression of JAK2, STAT3, and the downstream inflammatory cytokines IL-6, IL-17, and IL-23 in the ankle joint tissues of CIA rats. However, it should be acknowledged that the current study design does not fully allow definitive discrimination between direct pharmacological inhibition of the JAK2/STAT3 pathway by YBD and adaptive changes secondary to the amelioration of arthritis severity—a complexity that arises from the inherent bidirectional interplay between the JAK/STAT pathway and the inflammatory microenvironment in RA.

Notably, the magnitude of reduction in JAK2 and STAT3 expression was markedly greater than that observed for the upstream cytokines IL-6 and IL-23 (Fig. **12** ). This disparity provides indirect evidence suggesting that YBD may exert regulatory effects on the JAK2/STAT3 pathway that are at least partially independent of its suppression of upstream inflammatory cytokines. From the network pharmacology perspective, although bioactive compounds in YBD, such as quercetin and luteolin, were not predicted to directly target JAK2 or STAT3, they may act synergistically on multiple nodes upstream and downstream of the pathway. Moreover, given the multicomponent nature of YBD, it is plausible that different active constituents simultaneously target upstream inflammatory signals (*e.g.*, IL-6, IL-23) and the core kinases of the JAK/STAT pathway, thereby producing a dual inhibitory effect.

Collectively, the current data support the interpretation that the inhibitory effect of YBD on the JAK2/STAT3 pathway is at least partially independent of its regulation of upstream inflammatory cytokines. Nevertheless, this inference warrants further validation through *in vitro* cellular assays (*e.g.*, using JAK2-overexpressing models or combined intervention with specific inhibitors) and molecular interaction studies (*e.g.*, surface plasmon resonance, molecular docking). Future studies will focus on evaluating the direct inhibitory effects of YBD-containing serum or its active constituents on JAK2 phosphorylation in fibroblast-like synoviocytes to further elucidate the underlying molecular mechanisms.

 As described above, IL-6 functions as a key positive regulator of T-lymphocyte differentiation and inflammatory responses and also contributes directly to osteoclast activation and bone destruction. Accordingly, the observed reduction in IL-6 levels is likely to attenuate rheumatoid arthritis pathogenic processes and mitigate cartilage and bone degradation [71-73]. Concurrently, decreased IL-23 levels may limit the differentiation and maintenance of Th17 cells, resulting in reduced IL-17 production and subsequent dampening of IL-17–mediated inflammatory cascades. This coordinated downregulation effectively suppresses the IL-23/IL-17 inflammatory axis, thereby alleviating downstream inflammatory responses. As key upstream activators of the JAK/STAT signaling pathway, reduced levels of IL-6 and IL-23 concurrently inhibit activation of the JAK2/STAT3 pathway, disrupting the self-amplifying inflammatory cycle and contributing to disease amelioration [74, 75]. Consistent with these molecular findings, comparative histopathological analysis of ankle joint sections from the Yibi Decoction group before and after treatment demonstrated marked preservation of cartilage surface integrity and a significant reduction in synovial inflammatory cell infiltration following intervention. However, residual pathological features, including disrupted cartilage tidemarks and mild hyperplasia of the synovial lining layer, were still observed following treatment (Fig. **7**). In addition, compared with the normal control group, residual deformities persisted in the interphalangeal joints of rats treated with Yibi Decoction. These pathological manifestations closely resemble the sequelae observed in human rheumatoid arthritis following synovial inflammation and progressive bone and cartilage destruction, underscoring the critical importance of early therapeutic intervention—particularly before the onset of irreversible structural damage to joint tissues. Notably, comprehensive Western blot analysis revealed that the magnitude of reduction in JAK2 and STAT3 expression was greater than that observed for IL-6, IL-17, and IL-23, indicating a disproportionate decrease. This finding raises the possibility that, in addition to indirectly suppressing JAK2/STAT3 signaling *via* the IL-23/IL-17 axis, Yibi Decoction might influence the expression of JAK2 and STAT3 through mechanisms that are partially independent of its effects on these upstream cytokines. However, direct evidence for a direct molecular interaction between YBD components and JAK2/STAT3 is lacking and requires further investigation. Such dual regulatory effects further attenuate activation of the JAK2/STAT3 signaling pathway, thereby contributing to the alleviation of joint inflammation in rheumatoid arthritis. Overall, the therapeutic efficacy of Yibi Decoction was numerically similar to that of methotrexate, although no direct statistical comparison was performed between the two treatment groups. (Fig. **14**).

## CONCLUSION

This integrated study demonstrates that Yibi Decoction (YBD) ameliorates rheumatoid arthritis through multi-component and multi-target mechanisms. Network pharmacology analysis predicted that key bioactive compounds, such as quercetin and luteolin, interact with RA-associated targets and signaling pathways. *In vivo* experimental validation further provided direct evidence that YBD effectively alleviates joint inflammation and pathological damage in collagen-induced arthritis (CIA) rats. Mechanistically, these therapeutic effects are mediated, at least in part, by suppression of the JAK2/STAT3 signaling pathway and its downstream pro-inflammatory cytokines. Collectively, these findings elucidate the mechanistic basis underlying the anti-rheumatic effects of YBD and provide a scientific rationale for its potential development as a therapeutic strategy for rheumatoid arthritis.

## LIMITATIONS OF THE STUDY

Although this study provides supportive evidence for the therapeutic potential of Yibi Decoction (YBD) in rheumatoid arthritis, several limitations should be acknowledged. First, the network pharmacology predictions, while informative, are inherently computational and therefore require further experimental validation to confirm the specific interactions between individual bioactive compounds and their predicted targets. In addition, the complex multi-component composition of YBD poses challenges in attributing the observed therapeutic effects to any single constituent, and the potential synergistic interactions among its components remain to be fully elucidated.

Second, although the collagen-induced arthritis (CIA) rat model is widely used and well established for studying rheumatoid arthritis, it does not fully recapitulate the chronicity, heterogeneity, and complexity of human RA. Moreover, this study was conducted over a defined experimental period, and therefore, the long-term efficacy and potential toxicity of Yibi Decoction (YBD) were not assessed. In addition, the experimental design evaluated only a single dosage of YBD; consequently, future studies are warranted to establish dose–response relationships and to optimize therapeutic dosing regimens.

Third, the mechanistic investigation in this study primarily focused on the JAK2/STAT3 signaling pathway and a selected set of inflammatory cytokines. Although this focus was supported by both bioinformatic predictions and experimental validation, the potential regulatory effects of Yibi Decoction (YBD) on other key pathways implicated in rheumatoid arthritis—such as NF-κB and MAPK signaling—cannot be excluded and warrant further investigation. In addition, the assessment of protein and mRNA expression at a single time point limits insight into the dynamic temporal regulation of these pathways during disease progression and treatment.

## Figures and Tables

**Fig. (1):**
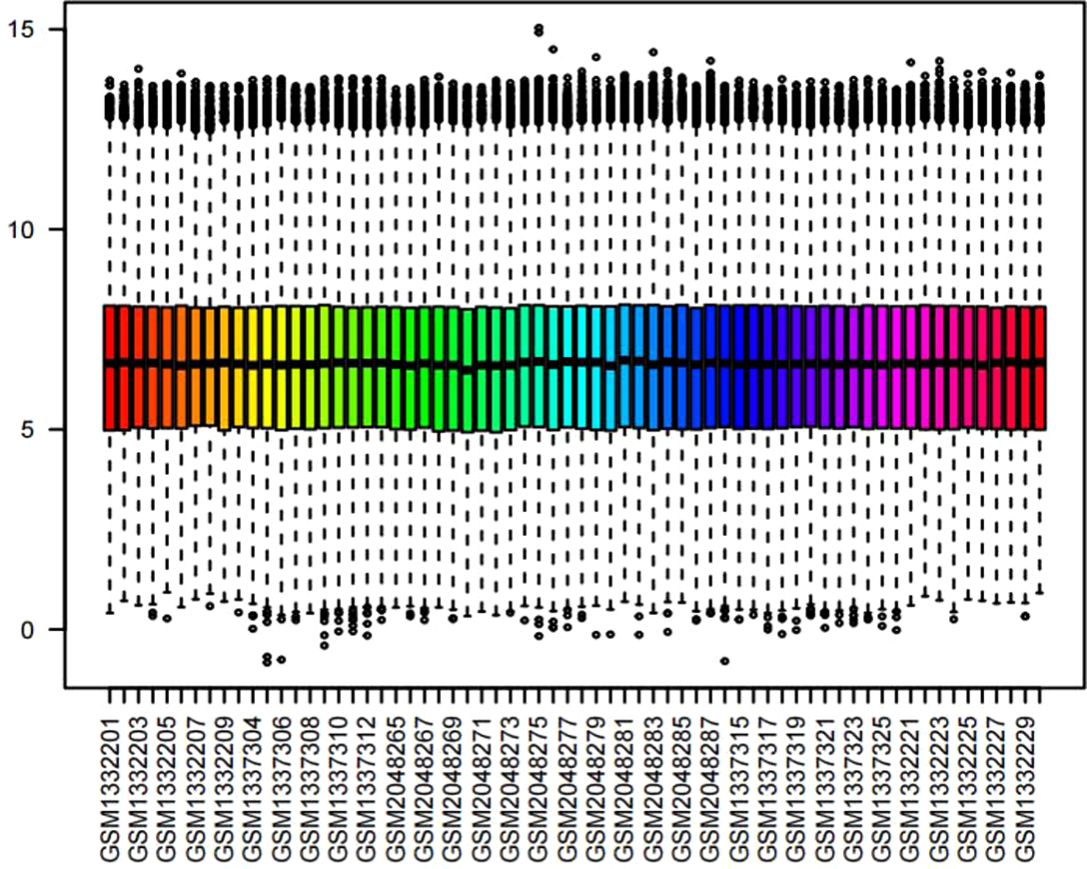
GEO dataset before batch effect elimination.

**Fig. (2):**
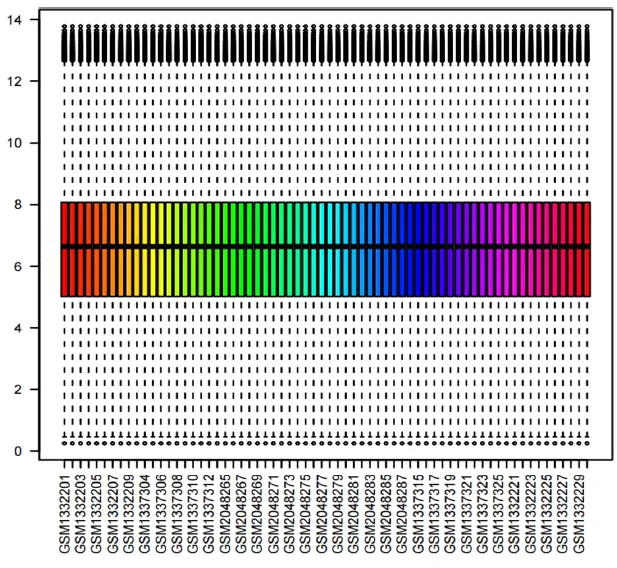
GEO dataset after batch effect elimination.

**Fig. (3):**
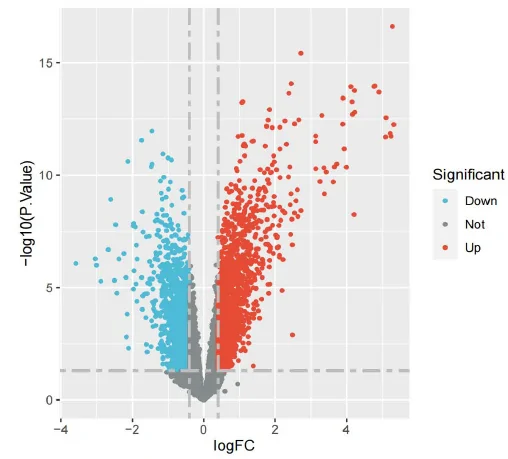
DEGs volcano plot.

**Fig. (4):**
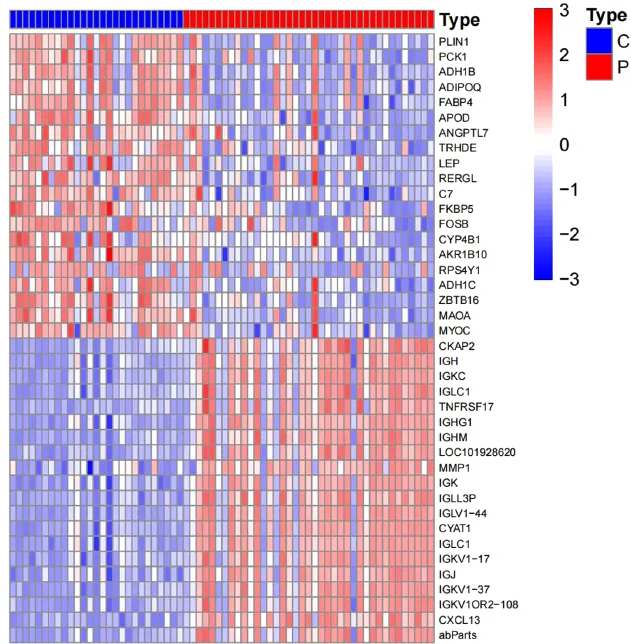
DEGs heatmap.

**Fig. (5):**
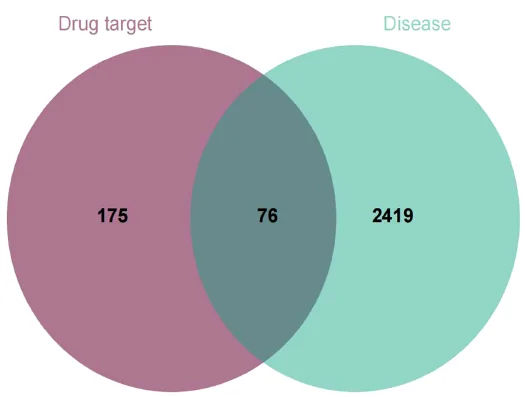
Venn diagram of drug target and RA.

**Fig. (6):**
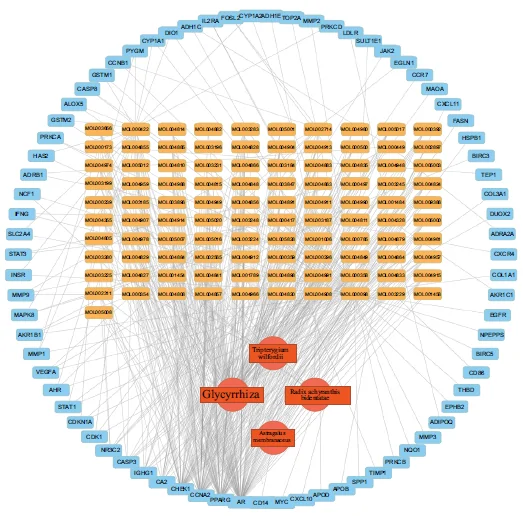
Herb-Compound-Target regulatory network.

**Fig. (7):**
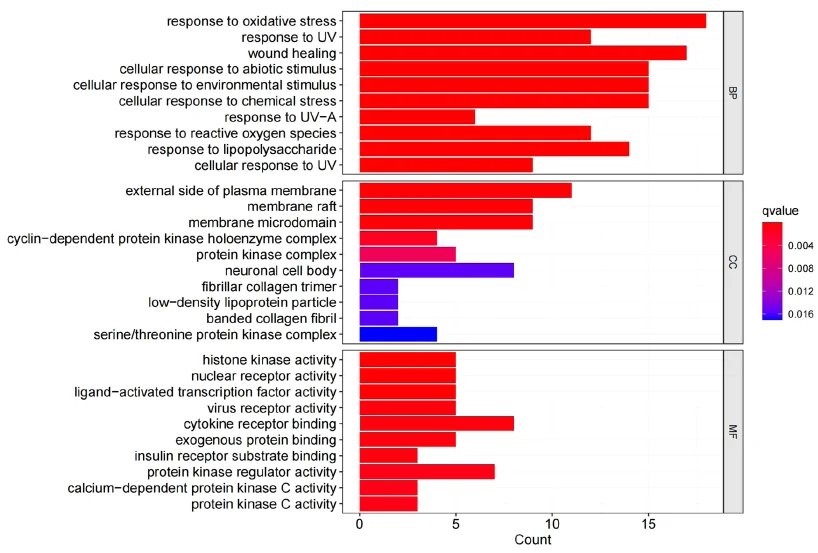
GO functional enrichment analysis.

**Fig. (8):**
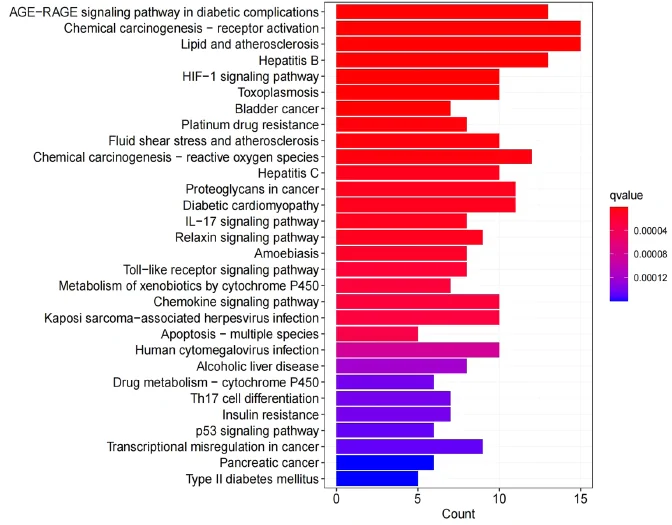
KEGG pathway enrichment analysis.

**Fig. (9):**
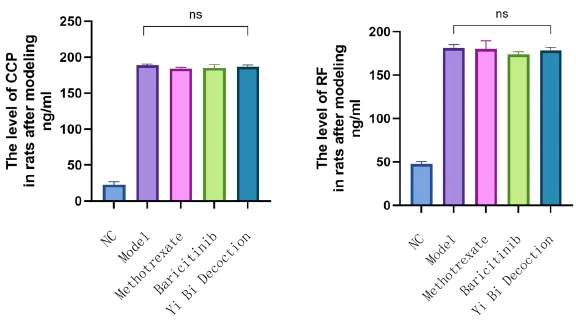
CCP and RF levels in the serum of *rats* at D28. In the modeling-treated group, both CCP and RF were significantly elevated. ns: The difference was not statistically significant.

**Fig. (10):**
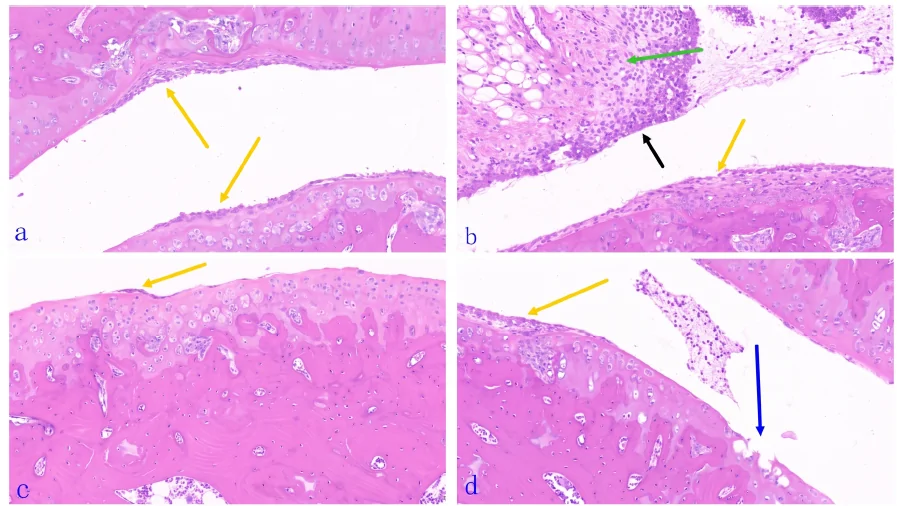
(**a**) model group, (**b**) methotrexate group, (**c**) baricitinib group, (**d**) Yibi Decoction group; Yellow arrows indicate irregular cartilage surfaces with pannus formation; green arrows denote synovial connective tissue hyperplasia; black arrows highlight infiltration of inflammatory cells, including lymphocytes and neutrophils; blue arrows mark cartilage surfaces. Hematoxylin-eosin staining, original magnification 200×.

**Fig. (11):**
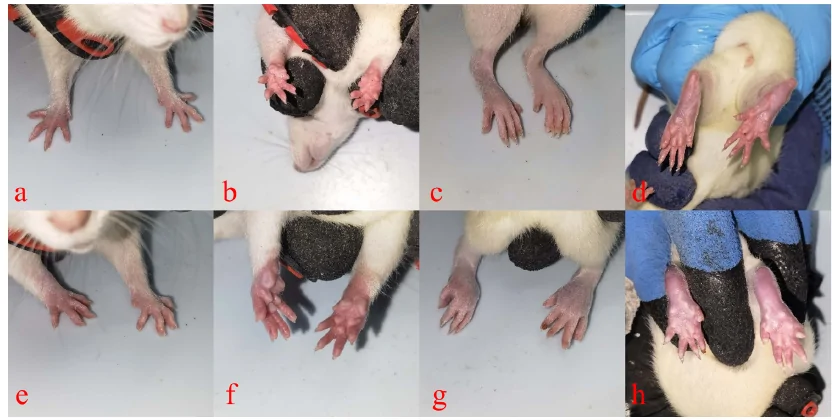
(**a-d**) Morphological presentation of rat forepaws and hindpaws of pre-modeling phase; (**e-h**) Morphological presentation of rat forepaws and hindpaws of post-modeling phase, showing evident redness and swelling in both fore and hind paws, with more pronounced manifestations in the hind paws.

**Fig. (12):**
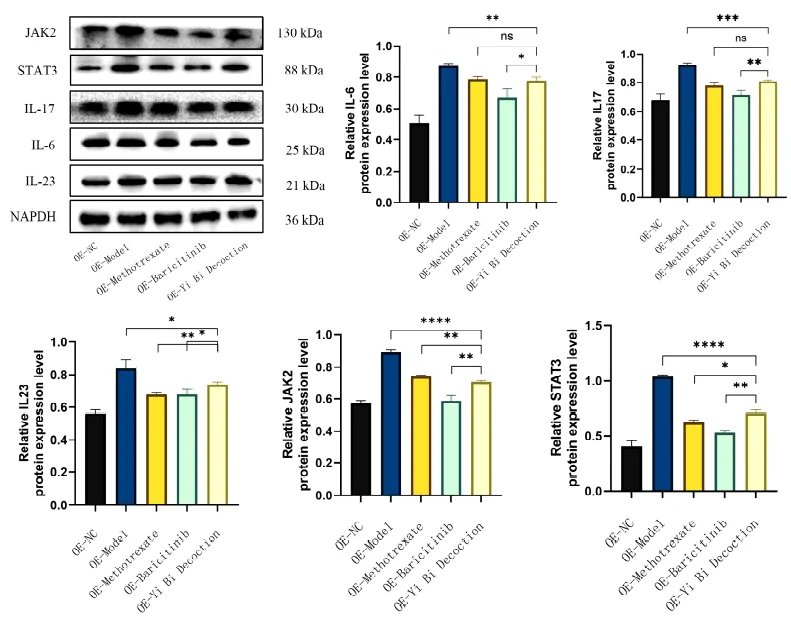
Western blot results after drug intervention. In the methotrexate, baricitinib, and Yibi decoction groups, IL-6, IL-17, IL-23, JAK2, and STAT3 all showed a downward trend compared with the non-intervened model group. ns: The difference was not statistically significant. *:*P* < 0.05; **:*P* < 0.01; ***:*P* < 0.001; ****:*P* < 0.0001.

**Fig. (13):**
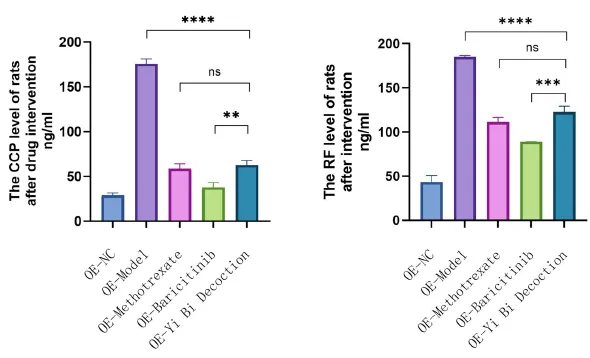
ELISA results after drug intervention. In the methotrexate, baricitinib, and Yibi decoction groups, CCP and RF were significantly lower than in the non-intervened model group. ns: The difference was not statistically significant. *:*P* < 0.05; **:*P* < 0.01; ***:*P* < 0.001; ****:*P* < 0.0001.

**Fig. (14):**
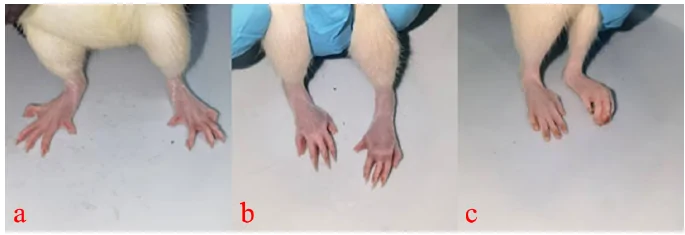
Morphological presentation of rat paw after Yibi Decoction intervention, (**a**) Pre-modeling phase, (**b**) Post-modeling phase, (**c**) Post-intervention with Yibi Decoction.

**Fig. (15):**
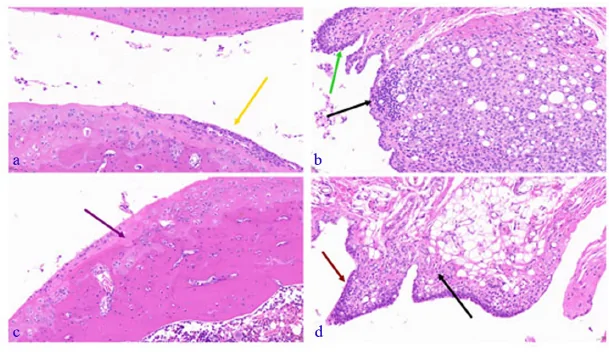
HE of rat joint tissues in the Yibi Decoction group before and after intervention. (**a-b**): Post-modeling phase; (**c-d**): Post-intervention with Yibi Decoction. Yellow arrows indicate irregular cartilage surfaces with pannus formation; green arrows denote synovial connective tissue hyperplasia; black arrows highlight infiltration of inflammatory cells, including lymphocytes and neutrophils; purple arrows mark disrupted tide lines with visible nuclear dissolution; brown arrows indicate hyperplasia of lining cells. Hematoxylin-eosin staining, original magnification 200×.

**Table 1:** Comparison of AI index scores in each group of rats.

**Groups**	**Index**	***F***	***P***
Blank group	0±0.00	130.22	<0.001
Model group	10.00±0.93*		
Methotrexate group	9.50±1.41*		
Baricitinib group	10.00±1.41*		
Yibi Decoction group	9.75±1.04*		

Note: *Compared with the blank group, *P*<0.05.

## Data Availability

The data that support the findings of this study are available from the corresponding author upon reasonable request.

## LIST OF ABBREVIATIONS

ACPAs: Anti-Citrullinated Protein Antibodies
AI: Arthritis Index
CC: Cellular Component
CCP: Cyclic Citrullinated Peptide
CIA: Collagen-Induced Arthritis
DEGs: Differentially Expressed Genes
DMARDs: Disease-Modifying Anti-Rheumatic Drugs
ELISA: Enzyme-Linked Immunosorbent Assay
FLS: Fibroblast-Like Synoviocytes
GEO: Gene Expression Omnibus
GO: Gene Ontology
HE: Hematoxylin and Eosin
IL: Interleukin
JAK: Janus Kinase
KEGG: Kyoto Encyclopedia of Genes and Genomes
MAPK: Mitogen-Activated Protein Kinase
MMPs: Matrix Metalloproteinases
NF-κB: Nuclear Factor Kappa-Light-Chain-Enhancer of Activated B Cells
NSAIDs: Non-Steroidal Anti-Inflammatory Drugs
RA: Rheumatoid Arthritis
RANKL: Receptor Activator of Nuclear Factor Kappa-B Ligand
RF: Rheumatoid Factor
STAT: Signal Transducer and Activator of Transcription
TCM: Traditional Chinese Medicine
TCMSP: Traditional Chinese Medicine Systems Pharmacology Database
TNF-α: Tumor Necrosis Factor-Alpha
VEGF: Vascular Endothelial Growth Factor
WB: Western Blot
YBD: Yibi Decoction

## References

[1] Cush J.J. (2022). Rheumatoid arthritis.. Rheum. Dis. Clin. North Am..

[2] Díaz-González F., Hernández-Hernández M.V. (2023). Rheumatoid arthritis.. Med. Clín. (Barc.).

[3] Di Matteo A., Bathon J.M., Emery P. (2023). Rheumatoid arthritis.. Lancet.

[4] Wu D., Luo Y., Li T., Zhao X., Lv T., Fang G., Ou P., Li H., Luo X., Huang A., Pang Y. (2022). Systemic complications of rheumatoid arthritis: Focus on pathogenesis and treatment.. Front. Immunol..

[5] Weyand C.M., Goronzy J.J. (2021). The immunology of rheumatoid arthritis.. Nat. Immunol..

[6] Ouboussad L., Burska A.N., Melville A., Buch M.H. (2019). Synovial tissue heterogeneity in rheumatoid arthritis and changes with biologic and targeted synthetic therapies to inform stratified therapy.. Front. Med. (Lausanne).

[7] Molteni E., Adinolfi A., Bondi V., Delvino P., Sakellariou G., Trentanni C., Ughi N., Pozzi M.R., Scirè C.A. (2024). Novel insights into the management of rheumatoid arthritis: One year in review 2024.. Clin. Exp. Rheumatol..

[8] Tanaka Y. (2023). Making a prescribing choice for rheumatoid arthritis: A focus on small molecule drugs *vs.* biologics for the most favourable patient outcome.. Expert Opin. Pharmacother..

[9] Betancourt B.Y., Biehl A., Katz J.D., Subedi A. (2018). Pharmacotherapy pearls in rheumatology for the care of older adult patients: Focus on oral disease-modifying antirheumatic drugs and the newest small molecule inhibitors.. Rheum. Dis. Clin. North Am..

[10] Yao H., Xiang L., Huang Y., Tan J., Shen Y., Li F., Geng F., Liu W., Li X., Gao Y. (2023). Guizhi Shaoyao Zhimu granules attenuate bone destruction in mice with collagen-induced arthritis by promoting mitophagy of osteoclast precursors to inhibit osteoclastogenesis.. Phytomedicine.

[11] Han L., Yan J., Li T., Shen P., Ba X., Lin W., Zhang R., Yang Y., Li Y., Li C., Huang Y., Qin K., Liu Y., Huang H., Zou L., Wang Y., Chen Z., Huang Y., Tu S. (2024). Wutou decoction alleviates arthritis inflammation in CIA mice by regulating Treg cell stability and Treg/Th17 balance *via* the JAK2/STAT3 pathway.. J. Ethnopharmacol..

[12] Feng Z., Fu L., Wang J., Zhu Y., He X., Zhou L., Zhou X. (2022). Efficacy of tripterygium glycosides (TG) in rheumatoid arthritis as a disease-modifying anti-rheumatic drug (DMARD) in combination with conventional DMARDs: A systematic review and meta-analysis of randomized controlled trials.. Pharmacol. Res..

[13] Jang S., Kwon E.J., Lee J.J. (2022). Rheumatoid arthritis: Pathogenic roles of diverse immune cells.. Int. J. Mol. Sci..

[14] Kubo S., Nakayamada S., Tanaka Y. (2023). JAK inhibitors for rheumatoid arthritis.. Expert Opin. Investig. Drugs.

[15] Azouz A.A., Saleh E., Abo-Saif A.A. (2020). Aliskiren, tadalafil, and cinnamaldehyde alleviate joint destruction biomarkers; MMP-3 and RANKL; in complete Freund’s adjuvant arthritis model: Downregulation of IL-6/JAK2/STAT3 signaling pathway.. Saudi Pharm. J..

[16] Wisłowska M. (2022). Comparison of treatment of severe rheumatoid arthritis patients with biological agents and JAK-STAT inhibitors. An observational study.. Reumatologia.

[17] Shao Y.H.J., Kuo T.T., Liao Y.J., Chen Y.T., Chen I.C., Kao C.M., Chen Y.J., Chen Y.M. (2026). Impact of drug-drug interaction of JAK inhibitors, CYP enzyme inhibitors and OAT3 inhibitors on persistence and infection rates in patients with autoimmune rheumatic diseases.. Joint Bone Spine.

[18] Jarneborn A., Kopparapu P.K., Jin T. (2025). The dual-edged sword: Risks and benefits of JAK inhibitors in infections.. Pathogens.

[19] González Mazarío R., Fragío Gil J.J., Ivorra Cortés J., Grau García E., Cañada Martínez A.J., González Puig L., Negueroles Albuixech R.M., Román Ivorra J.A. (2022). Real-world effectiveness and safety of JAK inhibitors in rheumatoid arthritis: A single-centre study.. Reumatol. Clín. (Engl. Ed.).

[20] Tran H.T.N., Ang K.S., Chevrier M., Zhang X., Lee N.Y.S., Goh M., Chen J. (2020). A benchmark of batch-effect correction methods for single-cell RNA sequencing data.. Genome Biol..

[21] Lin J., Sun A.R., Li J., Yuan T., Cheng W., Ke L., Chen J., Sun W., Mi S., Zhang P. (2021). A three-dimensional co-culture model for rheumatoid arthritis pannus tissue.. Front. Bioeng. Biotechnol..

[22] Zhang Y., He X., Yin D., Zhang Y. (2025). Redefinition of synovial fibroblasts in rheumatoid arthritis.. Aging Dis..

[23] Zhang Y., Wu Y., Yang H., Liu X., Pan B., Ding C., Sun Y., Sun L. (2025). NETs promote invasive behavior of fibroblast-like synoviocytes through GPIbα in rheumatoid arthritis.. Front. Immunol..

[24] Hoshino T., Tsuji K., Onuma H., Udo M., Ueki H., Akiyama M., Abula K., Katagiri H., Miyatake K., Watanabe T., Sekiya I., Koga H., Muneta T. (2018). Persistent synovial inflammation plays important roles in persistent pain development in the rat knee before cartilage degradation reaches the subchondral bone.. BMC Musculoskelet. Disord..

[25] Wu F., Gao J., Kang J., Wang X., Niu Q., Liu J., Zhang L. (2021). B cells in rheumatoid arthritis: Pathogenic mechanisms and treatment prospects.. Front. Immunol..

[26] Smith M.H., Gao V.R., Periyakoil P.K., Kochen A., DiCarlo E.F., Goodman S.M., Norman T.M., Donlin L.T., Leslie C.S., Rudensky A.Y. (2023). Drivers of heterogeneity in synovial fibroblasts in rheumatoid arthritis.. Nat. Immunol..

[27] Tsaltskan V., Firestein G.S. (2022). Targeting fibroblast-like synoviocytes in rheumatoid arthritis.. Curr. Opin. Pharmacol..

[28] Komatsu N., Takayanagi H. (2022). Mechanisms of joint destruction in rheumatoid arthritis — immune cell–fibroblast–bone interactions.. Nat. Rev. Rheumatol..

[29] Andreev D., Porschitz P., Weidner D., Song R., Weider M., Schett G., Gölz L., Bozec A. (2025). A multidimensional analysis of temporomandibular joint and ankle joint erosion in inflammatory arthritis.. Front. Immunol..

[30] Pu Y., Cheng R., Zhang Q., Huang T., Lu C., Tang Z., Zhong Y., Wu L., Hammock B.D., Hashimoto K., Luo Y., Liu Y. (2023). Role of soluble epoxide hydrolase in the abnormal activation of fibroblast-like synoviocytes from patients with rheumatoid arthritis.. Clin. Immunol..

[31] Brown P., Pratt A.G., Hyrich K.L. (2024). Therapeutic advances in rheumatoid arthritis.. BMJ.

[32] Ruscitti P., Cantarini L., Ciccia F., Conti F., Dagna L., Iannone F., Montecucco C., Giovanni P., Sfriso P., Giacomelli R. (2025). Managing the clinical heterogeneity of patients with Still's disease, from early diagnosis to timely treatment.. Autoimmun. Rev..

[33] Sakthiswary R., Uma Veshaaliini R., Chin K.Y., Das S., Sirasanagandla S.R. (2022). Pathomechanisms of bone loss in rheumatoid arthritis.. Front. Med..

[34] Cheng L., Rong X. (2025). Clinical application of biological agents in rheumatoid arthritis.. Transpl. Immunol..

[35] Ganeshalingam S., Wilson N.J., Ciciriello S., Antonipillai J., Achuthan A.A. (2025). Cellular interactions in maintaining an inflammatory microenvironment in rheumatoid arthritis.. Mol. Immunol..

[36] Perera J., Delrosso C.A., Nerviani A., Pitzalis C. (2024). Clinical phenotypes, serological biomarkers, and synovial features defining seropositive and seronegative rheumatoid arthritis: A literature review.. Cells.

[37] Li Z., Liu Y., Zhang Y., Zhang Z., Lei S., Shen H. (2025). NETs promote fibroblast-like synoviocytes producing IL-6 to enhance T follicular helper cell response in rheumatoid arthritis.. Clin. Rheumatol..

[38] Yue S., Fan J., Xie D., Cao C., Wang Z., Huang J., Qiu F., Yang X., He D., Lu A., Liang C. (2025). Unveiling the therapeutic potential: Targeting fibroblast-like synoviocytes in rheumatoid arthritis.. Expert Rev. Mol. Med..

[39] Navashenaq J.G., Shabgah A.G., Hedayati-Moghadam M., Ariaee N., Mohammadi H., Hemmatzadeh M., Azhdari S., Jamialahmadi T., Sathyapalan T., Sahebkar A. (2021). The role of myeloid-derived suppressor cells in rheumatoid arthritis: An update.. Life Sci..

[40] Kaymaz K., Beikler T. (2019). Th17 cells and the IL-23/IL-17 axis in the pathogenesis of periodontitis and immune-mediated inflammatory diseases.. Int. J. Mol. Sci..

[41] Schinocca C., Rizzo C., Fasano S., Grasso G., La Barbera L., Ciccia F., Guggino G. (2021). Role of the IL-23/IL-17 pathway in rheumatic diseases: An overview.. Front. Immunol..

[42] Banerjee S., Biehl A., Gadina M., Hasni S., Schwartz D.M. (2017). JAK-STAT signaling as a target for inflammatory and autoimmune diseases: Current and future prospects.. Drugs.

[43] Ciobanu D.A., Poenariu I.S., Crînguș L.I., Vreju F.A., Turcu-Stiolica A., Tica A.A., Padureanu V., Dumitrascu R.M., Banicioiu-Covei S., Dinescu S.C., Boldeanu L., Siloși I., Ungureanu A.M., Boldeanu M.V., Osiac E., Barbulescu A.L. (2020). JAK/STAT pathway in pathology of rheumatoid arthritis (Review).. Exp. Ther. Med..

[44] Arab H.H., Abd El-Aal S.A., Ashour A.M., El-Sheikh A.A.K., Al Khabbaz H.J., Arafa E.S.A., Mahmoud A.M., Kabel A.M. (2022). Targeting inflammation and redox perturbations by lisinopril mitigates Freund’s adjuvant-induced arthritis in rats: Role of JAK-2/STAT-3/RANKL axis, MMPs, and VEGF.. Inflammopharmacology.

[45] Nishimura R., Hata K., Takahata Y., Murakami T., Nakamura E., Ohkawa M., Ruengsinpinya L. (2020). Role of signal transduction pathways and transcription factors in cartilage and joint diseases.. Int. J. Mol. Sci..

[46] Min H.K., Won J.Y., Kim B.M., Lee K.A., Lee S.J., Lee S.H., Kim H.R., Kim K.W. (2020). Interleukin (IL)-25 suppresses IL-22-induced osteoclastogenesis in rheumatoid arthritis *via* STAT3 and p38 MAPK/IκBα pathway.. Arthritis Res. Ther..

[47] Zhao F., Hu Z., Li G., Liu M., Huang Q., Ai K., Cai X. (2025). Angiogenesis in rheumatoid Arthritis: Pathological characterization, pathogenic mechanisms, and nano-targeted therapeutic strategies.. Bioact. Mater..

[48] Chen L., Liang L. (2025). Osteoimmunology of osteoporosis in rheumatoid arthritis: Emerging mechanisms and therapeutic implications.. Int. J. Gen. Med..

[49] Sciacca E., Surace A.E.A., Alaimo S., Pulvirenti A., Rivellese F., Goldmann K., Ferro A., Latora V., Pitzalis C., Lewis M.J. (2022). Network analysis of synovial RNA sequencing identifies gene-gene interactions predictive of response in rheumatoid arthritis.. Arthritis Res. Ther..

[50] Geng Q., Xu J., Cao X., Wang Z., Jiao Y., Diao W., Wang X., Wang Z., Zhang M., Zhao L., Yang L., Deng T., Fan B., Xu Y., Jia L., Xiao C. (2024). PPARG-mediated autophagy activation alleviates inflammation in rheumatoid arthritis.. J. Autoimmun..

[51] Xu K., Qin X., Zhang Y., Yang M., Zheng H., Li Y., Yang X., Xu Q., Li Y., Xu P., Wang X. (2023). *Lycium ruthenicum* Murr. anthocyanins inhibit hyperproliferation of synovial fibroblasts from rheumatoid patients and the mechanism study powered by network pharmacology.. Phytomedicine.

[52] Choo M.Z.Y., Chua J.A.T., Lee S.X.Y., Ang Y., Wong W.S.F., Chai C.L.L. (2025). Privileged natural product compound classes for anti-inflammatory drug development.. Nat. Prod. Rep..

[53] Chen K., Chen X., Huang S., Zhou L., Zhou X., Lu Y., Feng Z. (2025). Therapeutic effects and mechanisms of Tripterygium wilfordii extracts in rheumatoid arthritis: A systematic review and meta-analysis of preclinical studies.. Sci. Rep..

[54] Zhao X., Kim Y.R., Min Y., Zhao Y., Do K., Son Y.O. (2021). Natural plant extracts and compounds for rheumatoid arthritis therapy.. Medicina (Kaunas).

[55] Wang S., Zhang J., Liu W., Zhang L., Li R., Wang Y., Li S., Li L., Li J., Zhou M., Wu Y., Ka Y., Tang Z., Wang W. (2025). Signal pathways in the treatment of Rheumatoid Arthritis with traditional Chinese medicine.. J. Ethnopharmacol..

[56] Nazakat L., Ali S., Summer M., Nazakat F., Noor S., Riaz A. (2025). Pharmacological modes of plant-derived compounds for targeting inflammation in rheumatoid arthritis: A comprehensive review on immunomodulatory perspective.. Inflammopharmacology.

[57] Behl T., Upadhyay T., Singh S., Chigurupati S., Alsubayiel A.M., Mani V., Vargas-De-La-Cruz C., Uivarosan D., Bustea C., Sava C., Stoicescu M., Radu A.F., Bungau S.G. (2021). Polyphenols targeting MAPK mediated oxidative stress and inflammation in rheumatoid arthritis.. Molecules.

[58] Qiu F., Xie D., Chen H., Wang Z., Huang J., Cao C., Liang Y., Yang X., He D.Y., Fu X., Lu A., Liang C. (2025). Generation of cytotoxic aptamers specifically targeting fibroblast-like synoviocytes by CSCT-SELEX for treatment of rheumatoid arthritis.. Ann. Rheum. Dis..

[59] Wang Z., Bai X., Zhang H., Yang M., Liu M., Nie T., Li T., Zhang M., Wang X., Wang J., Han J., Liu X. (2025). Targeting the ferroptosis pathway for rheumatoid arthritis: Molecular mechanisms and prospects for inhibitor development.. Front. Immunol..

[60] Zheng Y., Yan F., He S., Luo L. (2024). Targeting ferroptosis in autoimmune diseases: Mechanisms and therapeutic prospects.. Autoimmun. Rev..

[61] Zwicky P., Unger S., Becher B. (2020). Targeting interleukin-17 in chronic inflammatory disease: A clinical perspective.. J. Exp. Med..

[62] Wang S., Wan L., Zhang M., Yan D., Li F. (2025). New targets for immune inflammatory response in rheumatoid arthritis: Focus on the potential significance of N6-methyladenosine, ferroptosis and cuproptosis.. J. Inflamm. Res..

[63] Tang J., Liu W., Li Z., Shen C., Zhang L., Wang C., Wang F., Zai Z., Qian X., Hu W., Zhang X., Peng X., Xu Y., Chen F. (2025). Inhibition of ASIC1a reduces ferroptosis in rheumatoid arthritis articular chondrocytes *via* the p53/ NRF2 / SLC7A11 pathway.. FASEB J..

[64] Wang Y., Bao L., Liu M., Tang X., Liu H., Liang X., Liu Y. (2025). The role of the toll-like receptor signaling pathway in autoimmune diseases and treatment with traditional Chinese medicine: A literature review.. Endocr. Metab. Immune Disord. Drug Targets.

[65] Kalliolias G.D., Basdra E.K., Papavassiliou A.G. (2024). Targeting TLR signaling cascades in systemic lupus erythematosus and rheumatoid arthritis: An update.. Biomedicines.

[66] Zheng Y., Wei K., Jiang P., Zhao J., Shan Y., Shi Y., Zhao F., Chang C., Li Y., Zhou M., Lv X., Guo S., He D. (2024). Macrophage polarization in rheumatoid arthritis: Signaling pathways, metabolic reprogramming, and crosstalk with synovial fibroblasts.. Front. Immunol..

[67] Wang Y., Chen S., Du K., Liang C., Wang S., Owusu Boadi E., Li J., Pang X., He J., Chang Y. (2021). Traditional herbal medicine: Therapeutic potential in rheumatoid arthritis.. J. Ethnopharmacol..

[68] Mahto K., Kuwar O.K., Maloo A., Kalia N. (2025). Therapeutic potential of luteolin in neurodegenerative disorders: Targeting Nrf2, NFĸB, MAPK, and JAK-STAT pathways to combat neuroinflammation and apoptosis.. Inflammopharmacology.

[69] Lv P., Han P., Cui Y., Chen Q., Cao W. (2024). Quercetin attenuates inflammation in LPS-induced lung epithelial cells *via* the Nrf2 signaling pathway.. Immun. Inflamm. Dis..

[70] Pant T., Uche N., Juric M., Zielonka J., Bai X. (2024). Regulation of immunomodulatory networks by Nrf2-activation in immune cells: Redox control and therapeutic potential in inflammatory diseases.. Redox Biol..

[71] Yang M., Zhu L. (2024). Osteoimmunology: The crosstalk between T cells, B cells, and osteoclasts in rheumatoid arthritis.. Int. J. Mol. Sci..

[72] Jarlborg M., Gabay C. (2022). Systemic effects of IL-6 blockade in rheumatoid arthritis beyond the joints.. Cytokine.

[73] Yokota K. (2024). Osteoclast differentiation in rheumatoid arthritis.. Immunol. Med..

[74] Furtunescu A.R., Georgescu S.R., Tampa M., Matei C. (2024). Inhibition of the JAK-STAT pathway in the treatment of psoriasis: A review of the literature.. Int. J. Mol. Sci..

[75] Sisto M., Lisi S. (2023). Interleukin-23 involved in fibrotic autoimmune diseases: New discoveries.. J. Clin. Med..

